# Magnetic Anisotropy Modulation via van der Waals Gap Engineering in 2D Ferromagnet Fe_4_GeTe_2_

**DOI:** 10.1002/advs.202509941

**Published:** 2025-12-12

**Authors:** Weiran Xie, Guodong Wei, Tong Zhao, Hangtian Wang, Jing Li, Jihang Gao, Peiyuan Yu, Zili Wang, Fan Gao, Stéphane Mangin, Zhimei Sun, Weisheng Zhao, Jie Zhang, Tianxiao Nie

**Affiliations:** ^1^ Fert Beijing Institute School of Integrated Circuit Science and Engineering Beihang University Beijing 100191 China; ^2^ State Key Laboratory of Spintronics Hangzhou International Innovation Institute Beihang University Hangzhou 311115 China; ^3^ School of Materials Science and Engineering Beihang University Beijing 100191 China; ^4^ Policy and Economics Research Institute China Academy of Information and Communications Technology Beijing 100191 China; ^5^ Institut Jean Lamour Université de Lorraine Nancy F‐54506 France

**Keywords:** interface effect, magnetic anisotropy, spin reorientation, van der Waals ferromagnet, van der Waals gap

## Abstract

Magnetic anisotropy modulation is central to spintronics. 2D ferromagnetic materials (2D FMs), with their atomic‐level thickness, tunable electronic structures, and high sensitivity to external stimuli, provide unprecedented opportunities for precise magnetic control. Particularly, the van der Waals (vdW) gap holds promise for effective magnetic anisotropy modulation. However, the microscopic mechanisms remain elusive. Here, it is demonstrated that epitaxial growth of α‐Al_2_O_3_/Fe_4_GeTe_2_ induces a pronounced expansion of the vdW gap (up to 0.51 Å) at the interface, leading to a robust enhancement of in‐plane magnetic anisotropy (IMA) and suppression of the spin reorientation temperature (*T*_SR_) from 288 K to undetectable levels. This counterintuitive behavior contrasts with conventional thickness‐dependent perpendicular magnetic anisotropy (PMA). Combined experimental and theoretical analyses reveal that vdW gap expansion reduces Te p_x_/p_y_ orbital overlap, diminishing their contribution to magnetocrystalline anisotropy energy and suppressing PMA. These findings establish interface gap engineering as a novel route for tailoring magnetic anisotropy in 2D FMs, advancing the design of next‐generation spintronic devices.

## Graphical abstract

This study demonstrates a new route to control magnetic anisotropy in 2D ferromagnets via van der Waals gap engineering. In Fe_4_GeTe_2_, an interface‐induced vdW gap expansion is found to anomalously enhance in‐plane magnetic anisotropy while suppressing the spin reorientation transition. The underlying mechanism is elucidated, opening a new path for designing advanced 2D spintronic devices.

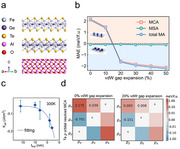

## 1 Introduction

Since the successful exfoliation of graphene,^[^ ^1^ ^]^ 2D materials have become an ideal platform for exploring novel physical properties and developing next‐generation electronic devices due to their unique quantum confinement effects,^[^ ^2^ ^]^ tunable electronic structures,^[^ ^3^, ^4^ ^]^ and excellent mechanical flexibility.^[^ ^5^ ^]^ As an important part of 2D materials, 2D ferromagnetic materials (2D FMs) have attracted much attention due to their potential applications in spintronics,^[^ ^6^ ^]^ magnetic storage,^[^ ^7^ ^]^ and other fields.^[^ ^8^, ^9^, ^10^ ^]^ Compared with traditional 3D magnetic materials, 2D FMs offer distinct advantages—most notably atomic‐scale thickness^[^ ^11^ ^]^ and readily tunable properties^[^ ^12^ ^]^—providing an ideal platform for probing emergent magnetic phenomena and enabling the development of next‐generation magnetic devices. For example, by changing the number of layers,^[^ ^13^ ^]^ stress,^[^ ^14^, ^15^ ^]^ doping,^[^ ^16^, ^17^ ^]^ interface effects,^[^ ^18^ ^]^ and other methods,^[^ ^19^, ^20^, ^21^ ^]^ the magnetism can be effectively tuned, thereby achieving precise control of the performance of magnetic devices.

Magnetic anisotropy is the core property that determines the feasibility of magnetic devices practical applications. As a key factor in resisting thermal fluctuations and maintaining magnetic order, magnetic anisotropy not only determines the direction of the easy magnetization axis of the material, but also directly affects the dynamic behavior closely related to device performance, such as domain wall motion^[^ ^22^, ^23^ ^]^ and skyrmion stability.^[^ ^24^ ^]^ Current strategies for modulating magnetic anisotropy in 2D FMs, such as doping,^[^ ^25^ ^]^ interface engineering,^[^ ^26^, ^27^ ^]^ and electric field control,^[^ ^28^ ^]^ are hindered by critical limitations. Doping may induce lattice defects and nonmagnetic phases that degrade magnetic properties. Interface engineering suffers heterostructure fabrication process and instability that restrict practical applications. Meanwhile, electric field modulation demands high operating voltages and ultrathin dielectric layers, which compromise energy efficiency and long‐term reliability. In addition to the external control methods mentioned above, strain engineering, which modifies the intrinsic structure of the material, has also been widely demonstrated as an effective strategy for tuning the magnetism of 2D materials. For example, in various 2D FMs such as CrI_3_,^[^ ^29^ ^]^ Cr_2_Ge_2_Te_6_,^[^ ^30^ ^]^ and VSe_2_,^[^ ^31^ ^]^ researchers have successfully achieved effective control over their magnetic properties using strain. The common physical basis for these works is that strain influences the orbital hybridization and spin–orbit coupling that determine magnetism by altering the crystal structure. In this context, the van der Waals (vdW) gap, as an intrinsic structural feature of 2D materials, provides a unique and intrinsic pathway to achieve this type of structural control. Its core principle is that altering the vdW gap directly adjusts the orbital overlap between interlayer atoms. The degree of this overlap, in turn, determines the material's electronic band structure and local crystal field environment, ultimately governing the strength of the spin–orbit coupling.^[^ ^32^ ^]^ Through applying strain to engineer the vdW gap, we anticipate the ability to directly manipulate crystal field symmetry and anisotropic exchange interactions. This would allow for deterministic control over magnetism at a low energy cost. Although the influence of the vdW gap on spin–orbit coupling and consequently on magnetism is understood in principle, how this effect specifically translates into the ability to control the key property of magnetic anisotropy is, from both experimental and theoretical standpoints, still not fully understood.

In recent years, iron‐based 2D FMs Fe_n_GeTe_2_ (*n* = 3–5) have been regarded as promising candidate systems for room‐temperature 2D FMs due to their high Curie temperature (*T*_C_) and strong spin–orbit coupling (SOC) effect.^[^ ^33^ ^]^ Recent work have demonstrated their significant practical potential, for instance, in efficient room‐temperature terahertz emission,^[^ ^9^, ^34^ ^]^ low‐pass filtering,^[^ ^10^ ^]^ and the construction of neural networks for digit recognition.^[^ ^35^ ^]^ Among them, and in contrast to Fe_3​_GeTe_2_​ which has strong perpendicular magnetic anisotropy but a lower *T*_C_ (≈220K),^[^ ^13^ ^]^ Fe_4_GeTe_2_ stands out as an ideal platform for probing vdW gap‐mediated magnetic anisotropy manipulation, given its high *T*_C_ (can exceed 550K) and moderate magnetic anisotropy.^[^ ^36^ ^]^ In this study, we report the experimental demonstration of vdW gap‐mediated magnetic anisotropy modulation in 2D FMs Fe_4_GeTe_2_. Systematic thickness‐dependent investigations reveal a striking c‐axis lattice expansion (≈0.3 Å) as the film thickness decreases from 16 to 4 nm. Atomic‐resolution HAADF‐STEM further identifies localized vdW gap widening (up to 0.51 Å) near interfacial regions, which correlates with suppressed spin reorientation temperature (*T*_SR_) and stabilized in‐plane magnetic anisotropy (IMA) across 20–400 K. Crucially, our first‐principles calculations establish a direct causal link between vdW gap expansion and electronic restructuring. The enlarged interlayer spacing weakens Te p_x_/p_y_ orbital hybridization, reducing their contribution to magnetocrystalline anisotropy (MCA). This orbital‐selective suppression of MCA energetically favors IMA dominance and explains the experimentally observed *T*_SR_ elimination. These results not only reveal the atomic‐scale mechanism of vdW gap‐driven magnetic anisotropy control in 2D FMs but also establish a framework for engineering anisotropy‐stable 2D FMs toward ultralow‐power spintronic memory and logic architectures.

## 2 Results and Discussion

### 2.1 Large‐Scale Growth and Characterization of Fe_4_GeTe_2_

**Figure 1:**
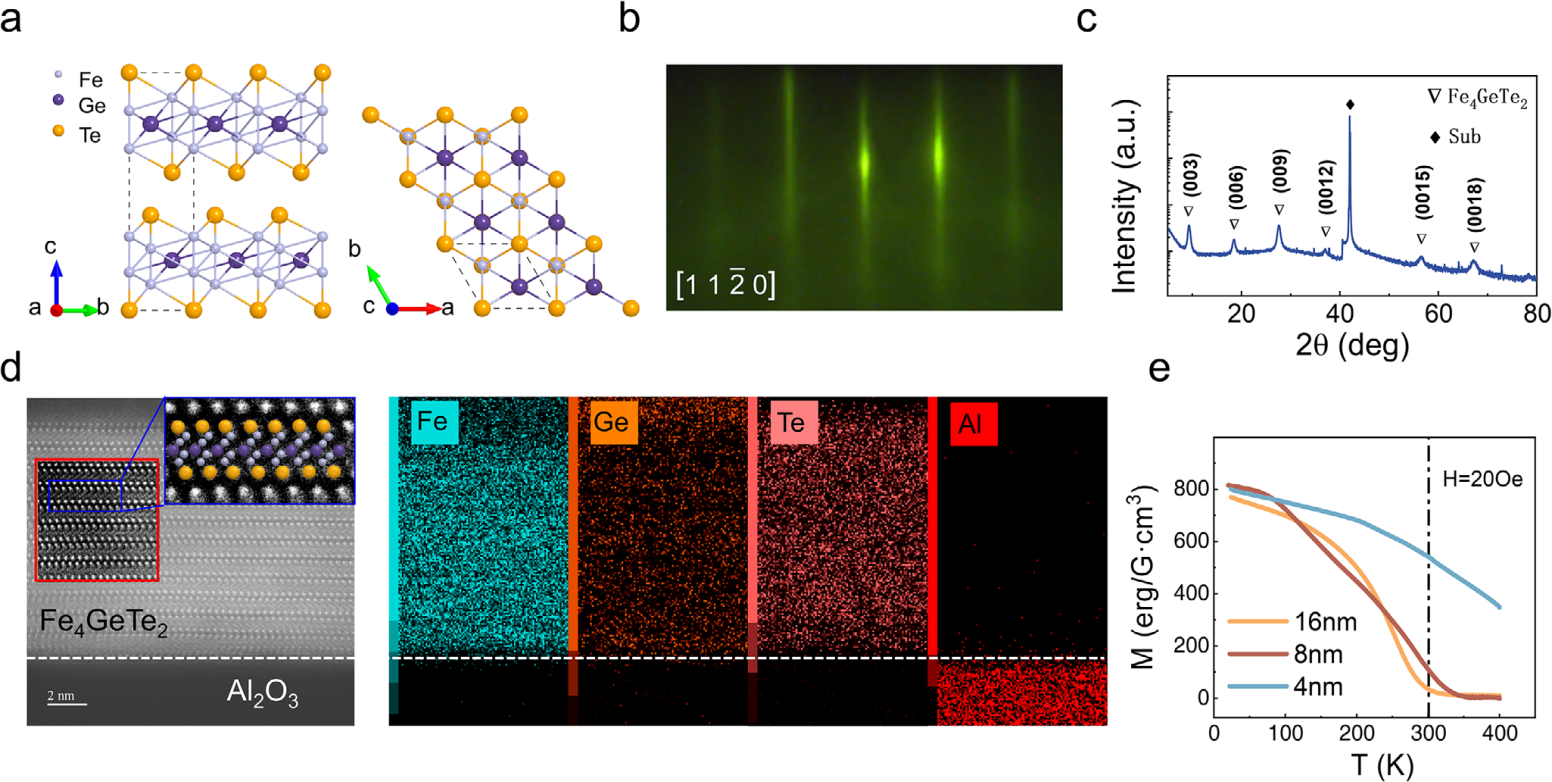
Structural and *T*_C_ characterization of Al_2_O_3_/Fe_4_GeTe_2_. a) Schematic illustration of the Fe_4_GeTe_2_ crystal structure. b) RHEED pattern of the Fe_4_GeTe_2_ along the [$11\bar{2}0$] direction, indicating high‐quality epitaxial growth. c) XRD scan of Fe_4_GeTe_2_ on Al_2_O_3_ substrate, displaying Fe_4_GeTe_2_ {003} family peaks. d) HAADF‐STEM image and corresponding EDS elemental maps of Fe, Ge, Te, and Al. Inset displays specific atomic distribution. e) Temperature‐dependent saturation magnetization curves of Fe_4_GeTe_2_ films with thicknesses of 4, 8, and 16 nm, measured under an applied magnetic field of 20 Oe.

Fe_4_GeTe_2_ belongs to the hexagonal crystal system with a space group of *R*‐3*m*. As shown in **Figure** **1**a, its crystal structure has Fe‐Fe diatomic pairs as the basic structural unit. These structural units are arranged alternately and obliquely along the ab plane, forming a 2D honeycomb lattice system with wave‐like features, and a Ge atom is embedded in the center of each hexagonal ring. At the monolayer scale, Fe_4_GeTe_2_ exhibits a typical layered vdW structure. The Fe_4_Ge heterometal layer dominated by covalent bonds is closely coordinated by the Te atom layer in a sandwich configuration (Te–Fe_4_Ge–Te), forming a 2D structure with a total (shape anisotropy (MSA) + MCA) anisotropy of both PMA and IMA type depending on temperature.^[^ ^36^ ^]^ The single‐layer thickness is approximately 9.7 Å, and the layers are weakly coupled by vdW gaps.

Considering that Fe_4_GeTe_2_ has good lattice matching with the α‐Al_2_O_3_(0001) substrate, it is beneficial for the growth of high‐quality thin films. In particular, our previous studies have shown that there is a significant interface coupling effect at the Fe_4_GeTe_2_/Al_2_O_3_ hetero‐interface, which can increase the *T*_C_ of the material to 550 K, breaking through the room temperature limit of traditional 2D FMs.^[^ ^37^ ^]^ This interface‐enhanced ferromagnetic order not only meets the basic requirements of high‐temperature magnetic order for practical applications of 2D spintronic devices, but, more importantly, it also provides an ideal platform for studying the critical phenomena of strongly correlated electron systems. Based on this, we choose Al_2_O_3_ (0001) as the substrate material for epitaxial growth of Fe_4_GeTe_2_. The substrate was pre‐treated by high‐temperature annealing (600 °C, 1 h, vacuum <1 × 10^−9^ Torr) to remove surface contaminants and remove the thermal oxide layer. Then, it was cooled down to 300 °C for epitaxial growth. During the growth process, reflection high‐energy electron diffraction (RHEED) was used to monitor the surface morphology in real time. As shown in Figure 1b, the sharp stripes displayed confirm the 2D layered growth mode. In view of the metastable properties of the Fe_4_GeTe_2_ system under environmental conditions,^[^ ^38^ ^]^ a 2 nm amorphous Ge capping layer was used for surface passivation after growth. X‐ray diffraction (XRD) analysis (Figure 1c) shows that the {003} family diffraction peaks corresponding to the Fe_4_GeTe_2_ crystal plane are clearly visible, and there are no impurity phases such as FeTe or GeTe, which confirms the high crystallization quality along the [001] orientation.^[^ ^39^ ^]^ In order to further understand the atomic‐scale structure, cross‐sectional characterization of the grown 16 nm sample was performed using high‐angle annular dark‐field scanning transmission electron microscopy (HAADF‐STEM) (Figure 1d). The interface between the Fe_4_GeTe_2_ layer and the Al_2_O_3_ substrate is clear, without any diffusion or amorphization regions. The thickness of each layer is 9.7 Å, which is consistent with previously reported data.^[^ ^37^ ^]^ In addition, energy dispersive X‐ray spectroscopy (EDS) analysis shows that the Fe, Ge, and Te elements are evenly distributed in the sample, confirming high‐quality epitaxial growth. A more detailed quantitative analysis across different regions of the sample is presented in Section S1 (Supporting Information). This supplementary analysis not only confirms the excellent homogeneity but also establishes the stoichiometric ratio of Fe:Ge:Te to be approximately 3.8:1:2.1 with a relative error of ≈4%.

Subsequently, magnetization versus temperature (M–T) curve measurements were performed on samples with different thicknesses. As shown in Figure 1e, it was found that the *T*_C_ of the Fe_4_GeTe_2_ showed a strong dependence on thickness. For the 16 nm sample, its *T*_C_ is about 280 K, while as the thickness is reduced to 8 and 4 nm, the *T*_C_ increases to 340 K and exceeds 400 K, respectively. This trend is highly consistent with the interface coupling enhancement mechanism in the Al_2_O_3_/Fe_4_GeTe_2_ system,^[^ ^37^ ^]^ indicating that interface engineering can effectively suppress spin fluctuations in the 2D limit, and further confirms the significant regulation effect of the interface on the magnetic properties of 2D FMs.

### 2.2 Investigation of Magnetic Anisotropy in Al_2_O_3_/Fe_4_GeTe_2_

**Figure 2:**
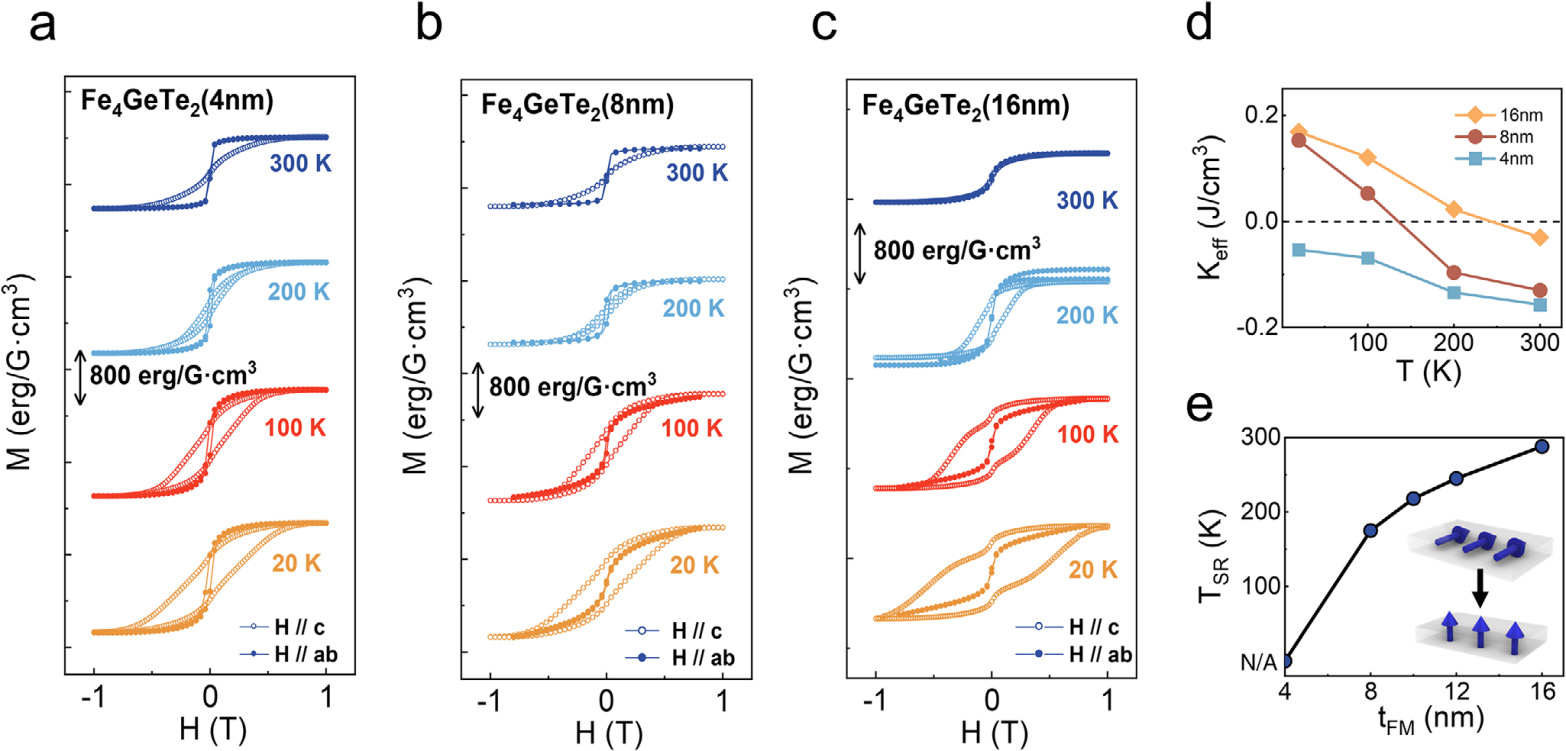
Anomalous magnetic anisotropy variation of Al_2_O_3_/Fe_4_GeTe_2_. a–c) M–H curves of Fe_4_GeTe_2_ with thicknesses of 4, 8, and 16 nm at different temperatures (20–300 K). The magnetic field was applied along the c‐axis (H//c) and the ab‐plane (H//ab) directions. d) Temperature dependence of the *K*_eff_ for Fe_4_GeTe_2_ films of varying thicknesses. e) *T*_SR_ as a function of ferromagnetic layer thickness (*t*_FM_). The inset illustrates the spin reorientation transition process.

Although the dimension‐enhanced effect of *T*_C_ in the Al_2_O_3_/Fe_4_GeTe_2_ system has been extensively studied,^[^ ^37^ ^]^ the modulation mechanism of its magnetic anisotropy remains an unknown area, and this research is crucial for the subsequent large‐scale preparation and application of this system. **Figure** **2**a–c shows typical hysteresis loops of 4, 8, and 16 nm thin films from 20 to 300 K. At 300 K, both the 4 nm (Figure 2a) and 8 nm (Figure 2b) films exhibit in‐plane magnetic anisotropy. Whereas the 16 nm film, due to its Curie temperature being below 300 K (Figure 2c), shows paramagnet‐like behavior. Based on fundamental magnetic theory, we can determine the magnetic anisotropy of a sample by comparing the remanence (*M*_r_)‐to‐saturation magnetization (*M*_s_) ratios (*M*_r_/*M*_s_) obtained from hysteresis loops measured with the magnetic field applied in the in‐plane and out‐of‐plane directions, respectively.^[^ ^40^, ^41^ ^]^ The *M*_r_​/*M*_s​_ ratios for samples of different thicknesses at various temperatures, obtained from their in‐plane and out‐of‐plane hysteresis loops, have been summarized in Section S2 (Supporting Information). We find that as the temperature decreases, the in‐plane *M*_r_/*M*_s_ ratio of the 4 nm Fe_4_GeTe_2_ sample is consistently larger than the out‐of‐plane ratio, which indicates that it maintains a stable IMA at all tested temperatures. In contrast, the thicker films exhibit a transition towards perpendicular magnetic anisotropy (PMA): the 16 nm Fe_4_GeTe_2_ shows PMA characteristics around 200 K, while the 8 nm Fe_4_GeTe_2_ transitions to being PMA‐dominated at 100 K. When the temperature is lowered to 20 K, the trend of IMA strengthening with decreasing thickness becomes more pronounced. Although the out‐of‐plane coercivity (H_C_) of the 4 nm sample increases to approximately 160 mT with decreasing temperature, its in‐plane to out‐of‐plane remanence ratio still indicates that the easy magnetization axis preferentially lies in‐plane. Conversely, the 8 and 16 nm films possess strong PMA at 20 K. This significant, thickness‐dependent evolution of magnetic anisotropy strongly suggests a modulating role of interface effects on magnetism, particularly causing the magnetic properties near the interface to differ from those of the bulk. Notably, the slight step feature observed in the out‐of‐plane magnetization curve of the 16 nm Fe_4_GeTe_2_ at low temperatures may stem from this magnetic inhomogeneity within the thicker film—that is, the region near the interface might exhibit IMA due to interface effects, while the bulk portion may retain different magnetic characteristics, leading to a step‐wise reversal of magnetic moments at different critical fields. The exact physical mechanism for this phenomenon will be elucidated later.

To quantify the magnetic anisotropy energy (MAE), we calculated the effective magnetic anisotropy constant (*K*_eff_) using the following formula:^[^ ^42^ ^]^

(1) $$K_{\mathrm{eff}}=\frac{H_{\mathrm{Sat}}\cdot M_{S}}{2}\;$$

where *H*_Sat_ is the saturation field along the hard axis, and *M*_s_ is the saturation magnetization. The sign of *K*_eff_ is then assigned by convention^[^ ^43^ ^]^: a positive sign indicates PMA, while a negative sign signifies IMA. Based on the determination method described above—comparing the in‐plane and out‐of‐plane *M*_r_/*M*_s_ ratios—we assign a sign to our calculated values. As shown in Figure 2d, through the calculation of the *K*_eff_, it can be concluded that at room temperature, the *K*_eff_ values for the 16, 8, and 4 nm samples are −0.04, −0.13, and −0.16 J cm^−3^, respectively. Although the *K*_eff_ remains negative as the thickness decreases, its absolute value increases, indicating an enhancement of IMA, which is consistent with our analysis above. Previous studies on Fe_4_GeTe_2_ prepared by mechanical exfoliation have well established that their PMA exhibits a significant enhancement with decreasing thickness.^[^ ^36^ ^]^ This is widely recognized as a typical manifestation of the dimensionality effect.^[^ ^44^, ^45^ ^]^ This effect describes that as the thickness of the film decreases, the broken inversion symmetry at the interface gives rise to a strong interfacial anisotropy contribution, which in turn leads to an enhancement of PMA. However, the trend observed in the Al_2_O_3_/Fe_4_GeTe_2_ system is completely opposite to the conventional expectations of the dimensionality effect. This phenomenon strongly suggests that interfacial effects introduce new and potentially dominant contributions to the overall magnetic anisotropy.

Evolution of the total anisotropy from IMA to PMA observed in the 8 and 16 nm samples is called spin reorientation transition.^[^ ^46^, ^47^, ^48^ ^]^ The origin of spin reorientation transition typically lies in the complex competition between MCA and MSA, where MCA primarily originates from the material's intrinsic crystal structure and the spin–orbit coupling (SOC) effect of electrons.^[^ ^49^ ^]^ Its strength is relatively sensitive to temperature changes and is a key factor driving the formation of low‐temperature PMA. Conversely, MSA is mainly contributed by the demagnetizing field effect arising from the thin film geometry. Its temperature dependence is weaker, and it generally tends to align the magnetization vector parallel to the film plane.^[^ ^50^ ^]^ When the temperature is lowered to a certain value, if the enhancement of MCA is sufficient to overcome the influence of MSA, the system will undergo a spin reorientation from IMA to PMA.^[^ ^47^ ^]^ This temperature is also known as the spin reorientation temperature (*T*_SR_). In mechanically exfoliated Fe_4_GeTe_2_, *T*_SR_ significantly increases from 110 K (bulk) to 200 K (7 layers) as the thickness decreases,^[^ ^36^ ^]^ further corroborating the traditional dimensionality effect where PMA is enhanced with decreasing thickness. In stark contrast to this behavior observed in mechanically exfoliated Fe_4_GeTe_2_, our experimental results for the Al_2_O_3_/Fe_4_GeTe_2_ reveal an opposite thickness dependence of the *T*_SR_. As shown in Figure 2d, the K_eff​_ of the 16 nm and 8 nm samples undergoes a sign reversal, indicating a spin reorientation transition. While this method provides an estimate for the T_SR_, we have performed additional M‐T measurements with the field applied in different orientations to determine the *T*_SR_ more precisely. From the intersection of the in‐plane and out‐of‐plane M–T curves (as detailed in Section S3, Supporting Information), we have accurately determined the *T*_SR_ for the 16 nm sample to be 288 K, and for the 8 nm sample to be 175 K. In contrast, the 4 nm sample maintains *K*_eff_ < 0 and shows no spin reorientation behavior within the entire tested temperature range (20–400 K), confirming its stable IMA. To systematically reveal the thickness dependence of *T*_SR_, we further measured the *T*_SR_ of five samples with different thicknesses (Figure 2e). The experimental data show that when the thickness is reduced from 16 to 4 nm, *T*_SR_ systematically decreases from 288 K to unobservable (N/A), and its decreasing rate significantly accelerates when the thickness is <8 nm. To corroborate the results from magnetic measurements, we also performed electrical transport characterization on samples of different thicknesses (as detailed in Section S4, Supporting Information). We utilized the anomalous Hall effect (AHE), a phenomenon where the *R*_xy_ is proportional to the perpendicular component of magnetization (*M*_Z_). This relationship allows us to probe the magnetic anisotropy electrically. Specifically, we analyzed the temperature dependence of the ratio between the remanent Hall resistance at zero field ($R_{\mathrm{xy}}^{0}$) and the saturation Hall resistance ($R_{\mathrm{xy}}^{\mathrm{sat}}$). This electrical ratio ($R_{\mathrm{xy}}^{0}$/$R_{\mathrm{xy}}^{\mathrm{sat}}$) serves as a proxy for the remanence ratio (*M*_r_/*M*_s_). The results show that the trend of this electrical ratio is highly consistent with our magnetic test results, further confirming the reliability of our experimental phenomena.

The observed suppression of spin reorientation, particularly the inability of MCA to induce it in the thinnest sample, strongly suggests that the Al_2_O_3_/Fe_4_GeTe_2_ interface critically influences the magnetic anisotropy competition, potentially via unconventional mechanisms. To elucidate the physical origin of this interfacial effect, further investigations into its impact on the local crystal structure and electronic states of Fe_4_GeTe_2_ are warranted.

### 2.3 Anomalous Evolution of Magnetic Anisotropy Driven by Lattice Distortion

**Figure 3:**
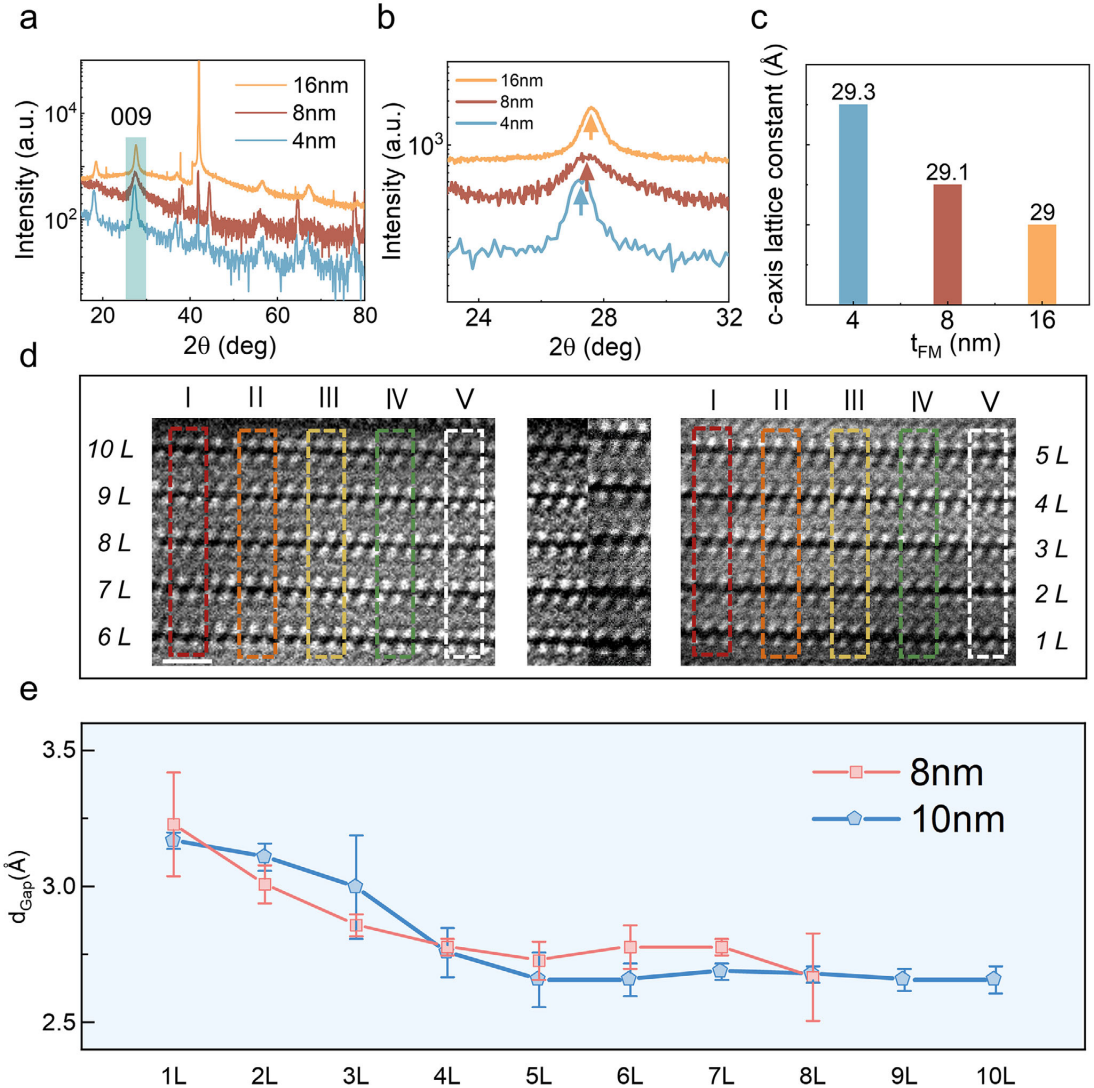
Structural evolution of Fe_4_GeTe_2_. a) XRD patterns of Fe_4_GeTe_2_ with different thicknesses. b) The enlarged image of the (009) diffraction peak. c) Trend of the c‐axis lattice constant of Fe_4_GeTe_2_ as a function of thickness, calculated from XRD results. d) HAADF‐STEM image of five layers near the interface (1L‐5L) and five layers away from the interface (6L‐10L) in 10 nm Fe_4_GeTe_2_. The middle image shows a detailed vdW gap comparison. Colored rectangles mark five selected analysis regions (I–V) for measuring the interlayer vdW gap. Scale bar: 1 nm. e) Average d_Gap_ as a function of layer number for Fe_4_GeTe_2_ of different thicknesses.

To reveal the physical origin of the anomalous thickness dependence of magnetic anisotropy in Fe_4_GeTe_2_, we first analyzed the crystal structure of samples with different thicknesses (4, 8, and 16 nm) using high‐resolution XRD. **Figure** **3**a shows the XRD patterns along the [001] direction, focusing on the shift of the (009) diffraction peak. It can be observed that as the thickness decreases, the (009) peak position of Fe_4_GeTe_2_ gradually shifts towards the low‐angle direction (as shown in Figure 3b), indicating that the c‐axis lattice constant significantly increases as the thickness decreases. Using the Bragg equation (2*d*sin*θ* = *nλ*, where *d* is the interplanar spacing, *θ* is the diffraction angle, and *λ* is the X‐ray wavelength), we calculated that the c‐axis lattice constant is 29 Å for the 16 nm sample, consistent with the reported value for bulk Fe_4_GeTe_2_, increasing to 29.1 Å for the 8 nm sample, and further increasing to 29.3 Å for the 4 nm sample. Figure 3c summarizes the trend of the c‐axis lattice constant change with thickness. Notably, the a‐axis lattice constant also exhibits a slight contraction trend with decreasing thickness (Section S5, Supporting Information), consistent with the previously reported in‐plane compressive strain induced by the Al_2_O_3_ substrate. Furthermore, XRD analysis on multiple samples with the same thickness confirmed the consistency of this trend and excluded inter‐sample variations (Section S6, Supporting Information).

To directly observe changes in the c‐axis lattice constant, we performed atomic‐resolution structural analysis using high‐angle annular dark‐field scanning transmission electron microscopy (HAADF‐STEM). Specifically, as shown in the left panel (labeled atomic layers 6L to 10L, representing the region far from the interface) and the right panel (labeled atomic layers 1L to 5L, representing the region near the interface) of Figure 3d, we selected and analyzed two distinct regions from a 10 nm Fe_4_GeTe_2_ sample that are significant for structural comparison. When comparing the interlayer spacing, we employed the alignment and cumulative measurement method intuitively demonstrated in the middle panel of Figure 3d. First, we precisely aligned the images using the bottommost atomic layers (1L and 6L) as baselines. Subsequently, by comparing the accumulated interlayer spacing across five atomic layers, potential errors associated with measuring individual interlayer spacings were minimized. The results clearly indicate that the thickness of individual Fe_4_GeTe_2_ atomic layers shows no significant change between the near‐interface and far‐interface regions, remaining essentially consistent. However, the accumulated total thickness over five atomic layers exhibits a distinct difference between these two regions. This indicates that the changes in lattice parameters do not originate from single‐layer structural distortions but are primarily due to variations in the vdW interlayer spacing.

Herein, we define *d*_Gap_ as the distance between adjacent Fe_4_GeTe_2_ atomic layers. For a quantitative analysis of *d*_Gap_ variations, we marked five selected analysis areas (labeled I, II, III, IV, and V) with rectangular boxes in the HAADF‐STEM images of 1L‐5L and 6L‐10L, respectively. Within these areas, we precisely measured *d*_Gap_, and the measurement results are summarized in **Table** **1**. Additionally, we measured the *d*_Gap_ variations in 8 nm Fe_4_GeTe_2_ using the same method, as shown in Section S7 (Supporting Information). Figure 3e clearly illustrates the trend of *d*_Gap_ variation as a function of atomic layer number in both 8 and 10 nm Fe_4_GeTe_2_. Analysis shows that the *d*_Gap_ exhibits a decreasing trend with increasing distance from the interface, eventually plateauing. Notably, the region near the interface demonstrates significant *d*_Gap_ expansion, indicating a substantial influence of the interface effect on interlayer spacing. Conversely, in the region far from the interface, the *d*_Gap_ variation is relatively minor, ruling out the possibility of overall structural distortion in the thin film. Considering the high sensitivity of Raman spectroscopy to interlayer coupling, we performed Raman measurements on samples with 4 and 16 nm Fe_4_GeTe_2_. A significant 18 cm^−1^ downward shift of the out‐of‐plane vibrational peak was observed in the 4 nm sample compared to the 16 nm sample, indicating a weakened interlayer coupling due to the vdW gap expansion.^[^ ^51^ ^]^ This result is consistent with our other experimental findings and is detailed in Section S8 (Supporting Information). These results collectively demonstrate that interface stress‐induced lattice relaxation is a key factor in the anomalous thickness dependence of magnetic anisotropy in Fe_4_GeTe_2_.

**Table 1:** Interlayer vdW gap measurements (Å).

	D_I_	D_II_	D_III_	D_IV_	D_V_	$\bar{D}$
10L	2.64	2.68	2.66	2.66	2.66	2.66
9L	2.64	2.68	2.65	2.67	2.66	2.66
8L	2.68	2.68	2.66	2.70	2.68	2.68
7L	2.67	2.71	2.69	2.69	2.69	2.69
6L	2.63	2.64	2.68	2.69	2.66	2.66
5L	2.62	2.62	2.60	2.64	2.76	2.66
4L	2.80	2.85	2.71	2.69	2.73	2.76
3L	3.10	3.06	3.02	3.02	2.81	3.0
2L	3.10	3.14	3.14	3.10	3.06	3.11
1L	3.18	3.18	3.18	3.18	3.14	3.17

### 2.4 First‐Principles Study of vdW Gap‐Engineered Magnetic Anisotropy Manipulation

**Figure 4:**
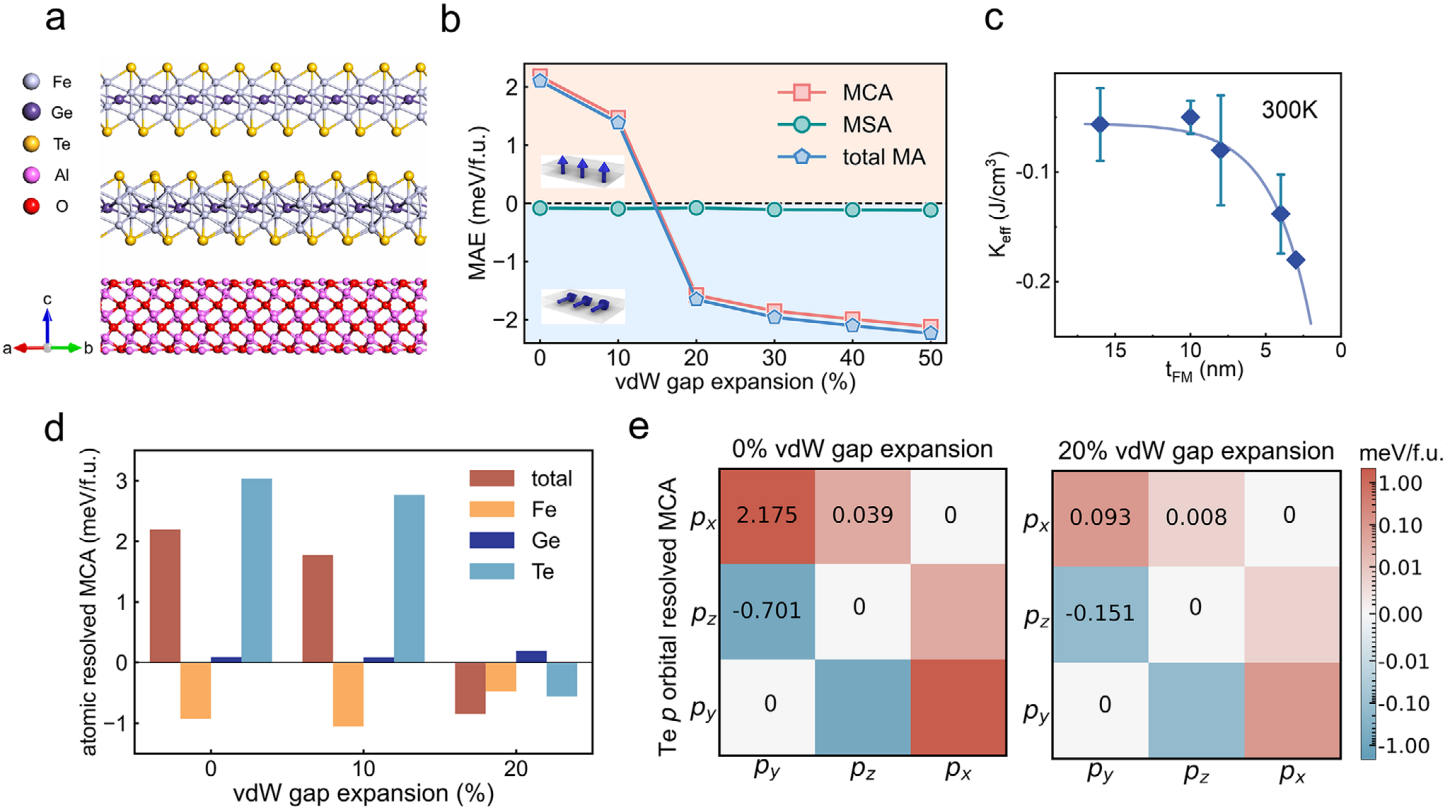
First‐principles calculations on the origin of anomalous magnetic anisotropy variation. a) Atomic structure of a two‐layer Fe_4_GeTe_2_ onto the sapphire substrate. b) Magnetic anisotropy as a function of vdW gap expansion in Fe_4_GeTe_2_. c) *K*_eff_ as a function of Fe_4_GeTe_2_ thickness. The solid line is a schematic curve that qualitatively shows the data trend. d) vdW gap expansion dependence of the atomic resolved MCA in Fe_4_GeTe_2_. e) Orbital–orbital magnetic anisotropy matrix of the Te p orbitals in Fe4GeTe2, under 0% and 20% vdW gap expansion. F.u. (formula unit) refers to a single‐layer unit cell of Fe_4_GeTe_2_.

To elucidate the correlation mechanism between the vdW gap expansion and the anomalous evolution of magnetic anisotropy, we conducted systematic research based on first‐principles calculations. Section S9 (Supporting Information) presents the first‐principles calculation results for bulk Fe_4_GeTe_2_. The results indicate that bulk Fe_4_GeTe_2_ exhibits PMA, consistent with experimental observations. Furthermore, to clarify the contribution of the interface to the anisotropy transition, we constructed a Fe_4_GeTe_2_/α‐Al_2_O_3_ heterostructure with a 2:$\sqrt{3}$ lattice‐matched interface (**Figure** **4**a). Theoretical simulations indicate an approximately 3% in‐plane tensile strain induced by the sapphire substrate in Fe_4_GeTe_2_, along with a significant, 47% expansion of the interfacial vdW gap. However, our experimental observations reveal these parameters to be 2% (as shown in Section S5, Supporting Information) and 20% (as reported earlier), respectively. This discrepancy arising from stress relaxation during the epitaxial growth process. After interface modulation, our first‐principles calculations show that the MAE exhibits negative values, with MCA and MSA components of ‐2.22 and ‐0.218 meV per f.u., respectively, indicating that spin reorientation leads to dominant IMA.

To directly probe the impact of vdW gap on MAE, we applied in‐plane and vdW gap strain stretching to Fe_4_GeTe_2_. As shown in Section S10 (Supporting Information), Fe_4_GeTe_2_ maintains PMA in the range of ‐4% to 4% in‐plane biaxial strain. In contrast, Figure 4b reveals that vdW gap stretching is key to controlling magnetic anisotropy. Within the applied strain range, MSA remains relatively stable (approximately ‐0.08 to ‐0.12 meV per f.u.), while MCA dominates the strain response. When the vdW gap stretching reaches a critical value of 20%, the sign of MCA changes from positive to negative (Figure 4b), driving the magnetization direction from out‐of‐plane to in‐plane. The room‐temperature measurement trend of *K*_eff_ for samples with different thicknesses in Figure 4c is consistent with theoretical predictions, further confirming the interfacial strain mechanism. Furthermore, considering that a reduction in thickness can also enhance MSA due to demagnetization effects, we have quantitatively analyzed the dependence of MSA on thickness. We found its change to be negligible compared to the large variation in MCA caused by the vdW gap expansion, thereby ruling out its dominant influence (see Section S11, Supporting Information, for details).

To understand the microscopic mechanism, we analyzed the atomic contribution to MCA by evaluating half of the SOC energy difference.^[^ ^52^ ^]^ Figure 4d shows the MCA contribution of each atom in Fe_4_GeTe_2_ under vdW gap expansion. Without applied strain, Te atoms, located near the vdW gap and possessing large SOC, make the dominant contribution. When a 10% expansion is applied, the MCA contribution of Te atoms significantly decreases to 2.76 meV per f.u., but remains positive. At 20% expansion, their contribution further weakens and becomes negative, leading to an overall change of MCA from positive to negative. In contrast, the contributions of Fe and Ge atoms to the strain‐induced MCA changes are an order of magnitude smaller than those of Te atoms, indicating that Te atoms dominate the anisotropy evolution induced by vdW gap expansion. Further analysis of the Te atom p‐orbital resolved SOC (Figure 4e) shows that the contribution of its p_x_ and p_y_ orbitals to MCA significantly decreases with the expansion of the vdW gap. In the equilibrium lattice, the MCA arising from SOC between the Te p_x_ and p_y_ orbitals is 2.175 meV per f.u. At 20% expansion, this value sharply decreases to 0.093 meV per f.u. This drastic reduction in orbital contribution is directly related to the increased spacing between Te atoms caused by the vdW gap expansion, which weakens the orbital hybridization intensity, thereby reducing the contribution to PMA.

## 3 Conclusion

In summary, we successfully synthesized high‐quality 2D FM Fe_4_GeTe_2_ on α‐Al_2_O_3_(0001) substrates via molecular beam epitaxy, and its anomalous magnetic anisotropy evolution behavior was systematically investigated. The experimental results show that the T_C_ significantly enhanced as the film thickness decreased, consistent with previous reports of interface‐enhanced ferromagnetism in this system. However, we also discovered an anomalous thickness dependence of magnetic anisotropy. Contrary to conventional expectations, the IMA strengthened with decreasing thickness, and the spin reorientation temperature was significantly suppressed, eventually vanishing in the ultrathin limit. Through high‐resolution X‐ray diffraction, HAADF‐STEM and first‐principles calculations, we revealed that this anomalous behavior originates from interface‐induced expansion of vdW gap at the α‐Al_2_O_3_/Fe_4_GeTe_2_ interface. As the film thickness decreases, the c‐axis lattice constant increases, leading to an expansion of the interatomic spacing between Te atom layers, which weakens the hybridization intensity between their p orbitals, thereby reducing the contribution to out‐of‐plane magnetic anisotropy. This interface‐induced expansion of vdW gap significantly alters the local bonding environment of Fe_4_GeTe_2_, thereby driving the anomalous evolution of magnetic anisotropy. Our research results achieved flexible regulation of the magnetic anisotropy of Fe_4_GeTe_2_ by vdW gap, not only revealing the crucial role of vdW gap in regulating the magnetic anisotropy of 2D FMs, but also providing new insights into understanding the size and interface effects of 2D FMs, providing a reference for the design and development of future 2D FMs.

## 4 Experimental Section

### Sample Preparation

High‐quality Fe_4_GeTe_2_ thin films were grown on a (0001) sapphire substrate in the molecular beam epitaxy (MBE) system. Before the growth, the substrate was annealed at 600 °C for an hour to remove the impurity layer and then cooled down to 300 °C. Elemental Fe, Ge, and Te solid sources were evaporated from standard Knudsen cells under an ultra‐high vacuum of 10^−10^ Torr. The beam flux rates of Fe, Ge, and Te were controlled at a ratio of 4:1:10. The deposition was in situ monitored by reflection high‐energy electron diffraction (RHEED). The growth rate was in situ monitored by rate monitoring equipment in real‐time. After growth, 2 nm Ge was deposited on the films as the protection layer from oxidation.

### Device Fabrication

The fabrication of Hall bar devices begins by defining the patterns on the film surface using a LaserWriter (Heidelberg DWL66+) at a wavelength of 375 nm. Subsequently, the pattern is transferred onto the film stack using an argon ion beam in a reactive ion etching (RIE) system to form the active region of the device. After etching, ultrasonic cleaning is performed sequentially in acetone, ethanol, and deionized water to completely remove the residual photoresist. To prevent performance degradation from exposure to the ambient atmosphere, an insulating dielectric layer is immediately deposited to encapsulate the device structure after its formation. Next, a second photolithography process defines the electrode pattern, and etching is performed to expose the top‐layer Ge as contact windows. Finally, the electrodes are fabricated through metal deposition and a subsequent lift‐off process. This fabrication method has been shown in previous studies to form good ohmic contacts.^[^ ^37^ ^]^

### High‐Resolution Transmission Electron Microscopy (HRTEM)

The cross‐section samples were prepared using the Helios 5UX focused ion beam system, and high‐resolution HAADF‐STEM images and EDS elemental mapping were subsequently acquired using the Thermo Fisher Scientific Spectra 300 TEM. The EDS analysis was conducted with an accelerating voltage of 200.0 kV and a probe current of 0.8 nA. Quantitative compositional data were acquired with a live time of 243 s to ensure sufficient signal‐to‐noise ratio.

### X‐Ray Diffraction (XRD)

After growth, the sample was characterized by the Bruker JV‐DX X‐ray diffractometer. The generator voltage and current of the setup were set to be 40 kV and 40 mA, respectively. The scanning range was 10°–60° with a step size of 0.03° and a time of 20 s per step.

### Superconducting Quantum Interference Device (SQUID)

The low‐temperature magnetization behavior of the samples was characterized in the temperature range of 20–400 K using the Quantum Design MPMS3 SQUID magnetometer, following established standard procedures to ensure accuracy and reliability. Magnetic field sweeps were conducted in the no‐overshoot, persistent mode to minimize hysteresis effects and improve measurement precision. Meanwhile, temperature sweeps were performed at a controlled rate of 3 K per minute to maintain thermal stability and uniformity across the sample. For each temperature point, the magnetic moment was calculated as the average of two independent scans, ensuring consistency and reducing potential errors in the measurement process.

### Raman Spectroscopy Measurements

Raman spectroscopy measurements were performed using a Horiba iHR550 spectrometer. A 532 nm laser was utilized as the excitation source. To prevent any laser‐induced thermal damage to the Fe_4_GeTe_2_ samples, the laser power was maintained at a low level, below 100 µW. Since the Al_2_O_3_ substrate does not exhibit any distinct characteristic peaks in the low wavenumber region of interest, no background subtraction was performed on the collected spectra.

### Theoretical Calculation

The first‐principles calculations were performed within the framework of density functional theory (DFT) using the Vienna ab initio simulation package (VASP).^[^ ^53^ ^]^ The projector augmented wave (PAW) pseudopotential^[^ ^54^ ^]^ was employed with a plane‐wave cutoff energy of 600 eV. Spin‐polarized local density approximation (LDA)^[^ ^55^ ^]^ was adopted to treat the exchange‐correlation interactions, as it has been proved to accurately describe the magnetic properties of Fe_3_GeTe_2_ and Fe_4_GeTe_2_.^[^ ^19^, ^56^ ^]^ To account for strong correlations, an effective Hubbard *U* = 3.5 eV was applied to the 3d electrons of Fe atoms.^[^ ^37^ ^]^ A vacuum region over 25 Å was introduced in the FGT/Al_2_O_3_ heterostructure to avoid interactions between neighboring slabs. All structures were relaxed with energy and force convergence criteria set to 10^−5 ^eV and 0.01 eV Å^−1^, respectively. The total magnetic anisotropy (MA) energy consists of magnetocrystalline anisotropy (MCA) energy and magnetic shape anisotropy (MSA) energy.^[^ ^57^, ^58^ ^]^ The MCA energy, derived from second‐order perturbation theory, is calculated by *E^MCA^* = ($E_{∥}^{SOC}-E_{⊥}^{SOC}$), where the magnetization is rotated from the in‐plane direction (∥) to the out‐of‐plane direction (⊥). The MSA arises from the magnetic dipole–dipole interaction and is expressed as follows:

(2) $$E^{D-D}=\frac{1}{2}\;\frac{\mu_{0}}{4\pi }\sum_{i\neq j}^{N}\frac{1}{r_{ij}^{3}}\left[M_{i}M_{j}-\frac{3}{r_{ij}^{2}}\left(M_{i}\cdot r_{ij}\right)\left(M_{j}\cdot r_{ij}\right)\right]$$

where μ_0_ is the vacuum permeability, *r_ij_* represents the vector connecting the Fe sites *i* and *j*, and *M* is the local magnetic moment. The MSA energy is calculated as *E^MSA^* = ($E_{∥}^{D}-E_{⊥}^{D}$).

## Conflict of Interest

The authors declare no conflicts of interest.

## Supporting information



Supporting Information

## Data Availability

The data that support the findings of this study are provided in the paper and Supporting Information file. Additional data related to this study are available from the corresponding author upon reasonable request.
