# Supplementary material for: Magnetic Anisotropy Modulation via van der Waals Gap Engineering in 2D Ferromagnet Fe4GeTe2
Source: Adv Sci (Weinh). 2025 Dec 12;13(11):e09941. doi: 10.1002/advs.202509941 (PMC12931250; doi:10.1002/advs.202509941)
Supplement: Supplementary file 1 — Supporting Information [file ADVS-13-e09941-s001.docx]

Supporting Information

Magnetic Anisotropy Modulation via van der Waals Gap Engineering in 2D Ferromagnet Fe_4_GeTe_2_

Weiran Xie, Guodong Wei*, Tong Zhao, Hangtian Wang, Jing Li, Jihang Gao, Peiyuan Yu, Zili Wang, Fan Gao, Stéphane Mangin, Zhimei Sun*, Weisheng Zhao, Jie Zhang*, Tianxiao Nie*

Section 1. Compositional Uniformity Analysis

To confirm the uniformity of the prepared samples, we performed a detailed elemental analysis on the 16nm Fe_4_GeTe_2_ using Transmission Electron Microscopy-Energy Dispersive X-ray Spectroscopy (TEM-EDS). This particular thickness was selected because it provides a sufficient cross-section to conduct a comparative analysis between the regions near and far from the interface. As shown in Figure S1 below, we selected two representative regions for a comparative analysis: Area 1, located in the bulk of the film far from the substrate interface, and Area 2, located near the film-substrate interface. EDS spectra were acquired from both regions. The quantitative analysis reveals that the atomic ratio in both regions is approximately Fe: Ge: Te ~ 3.8:1:2.1 with a relative error of ∼4%. This result provides strong evidence for the high chemical uniformity of our epitaxially grown films throughout their entire thickness.


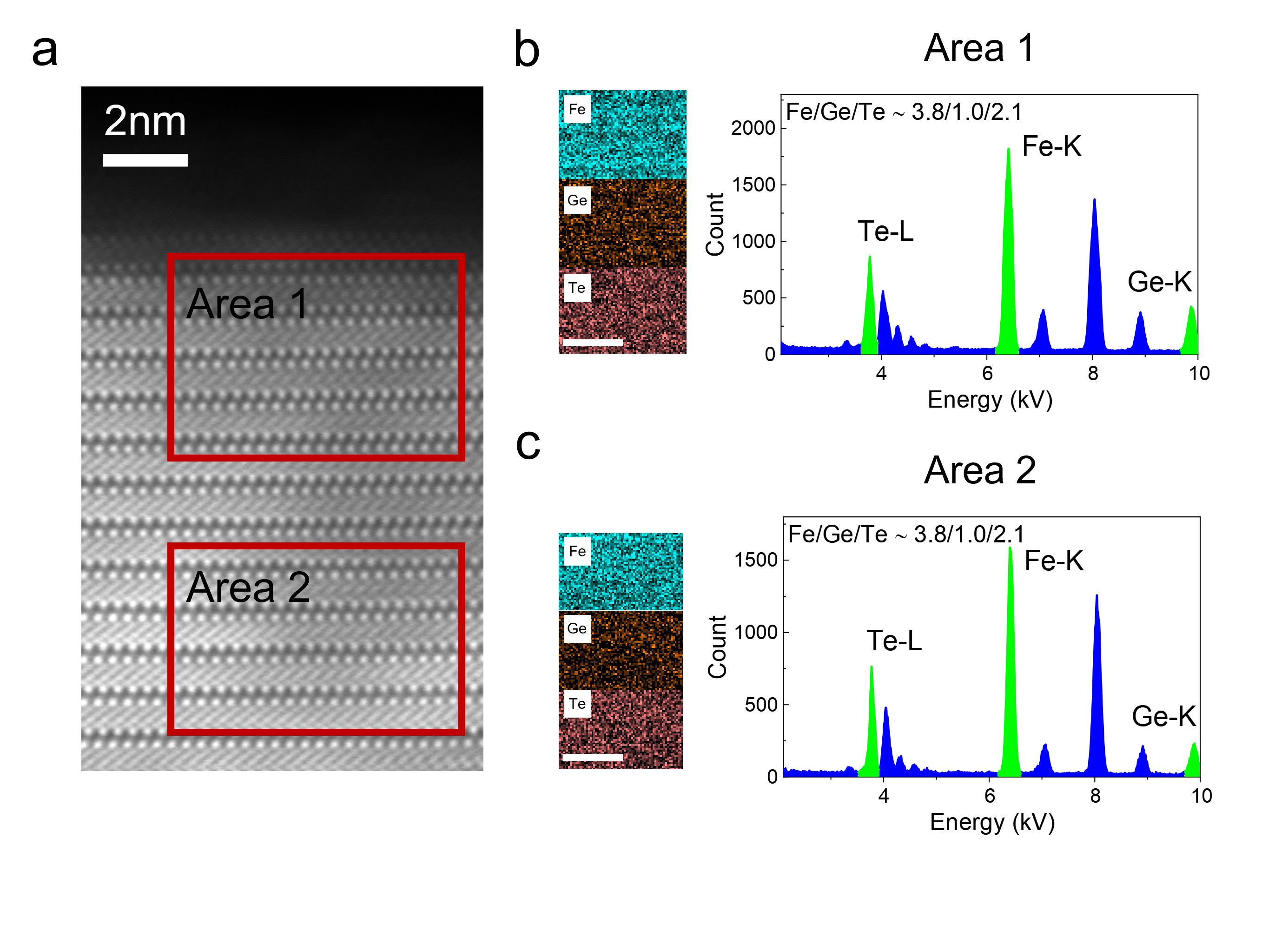


**Figure S1**. Compositional analysis of the Fe_4_GeTe_2_ film. a) High-Resolution Transmission Electron Microscopy (HRTEM) image of Fe_4_GeTe_2_. b, c) The EDS result verifies the 3.8:1.0:2.1 Fe:Ge:Te stoichiometric composition with the uniform element distribution map from Area 1 (far from the interface) and Area 2 (near the interface), respectively.

Section 2. Analysis of Magnetic Anisotropy via M_r_/M_s_ Ratios

To qualitatively analyze the evolution of magnetic anisotropy with temperature and thickness, we have summarized the individual remanence-to-saturation magnetization (M_r_/M_s_) ratios. These values were extracted from the in-plane and out-of-plane hysteresis loops measured for the 4 nm, 8 nm, and 16 nm thick samples at various temperatures. As shown in Figure S2, a comparison of the in-plane and out-of-plane M_r_/M_s_ ratios provides direct evidence for the orientation of the magnetic easy axis. For the 4 nm film, the in-plane M_r_/M_s_ ratio is consistently larger than the out-of-plane ratio across the entire temperature range from 20 K to 300 K, which confirms that the 4 nm sample maintains a stable in-plane magnetic anisotropy (IMA). For the 8 nm and 16 nm films, the out-of-plane M_r_/M_s_ ratio is dominant at low temperatures, confirming their perpendicular magnetic anisotropy (PMA). As the temperature increases, the out-of-plane ratio decreases while the in-plane ratio increases, with the curves crossing at the T_SR_​, indicating a transition to an in-plane easy axis.


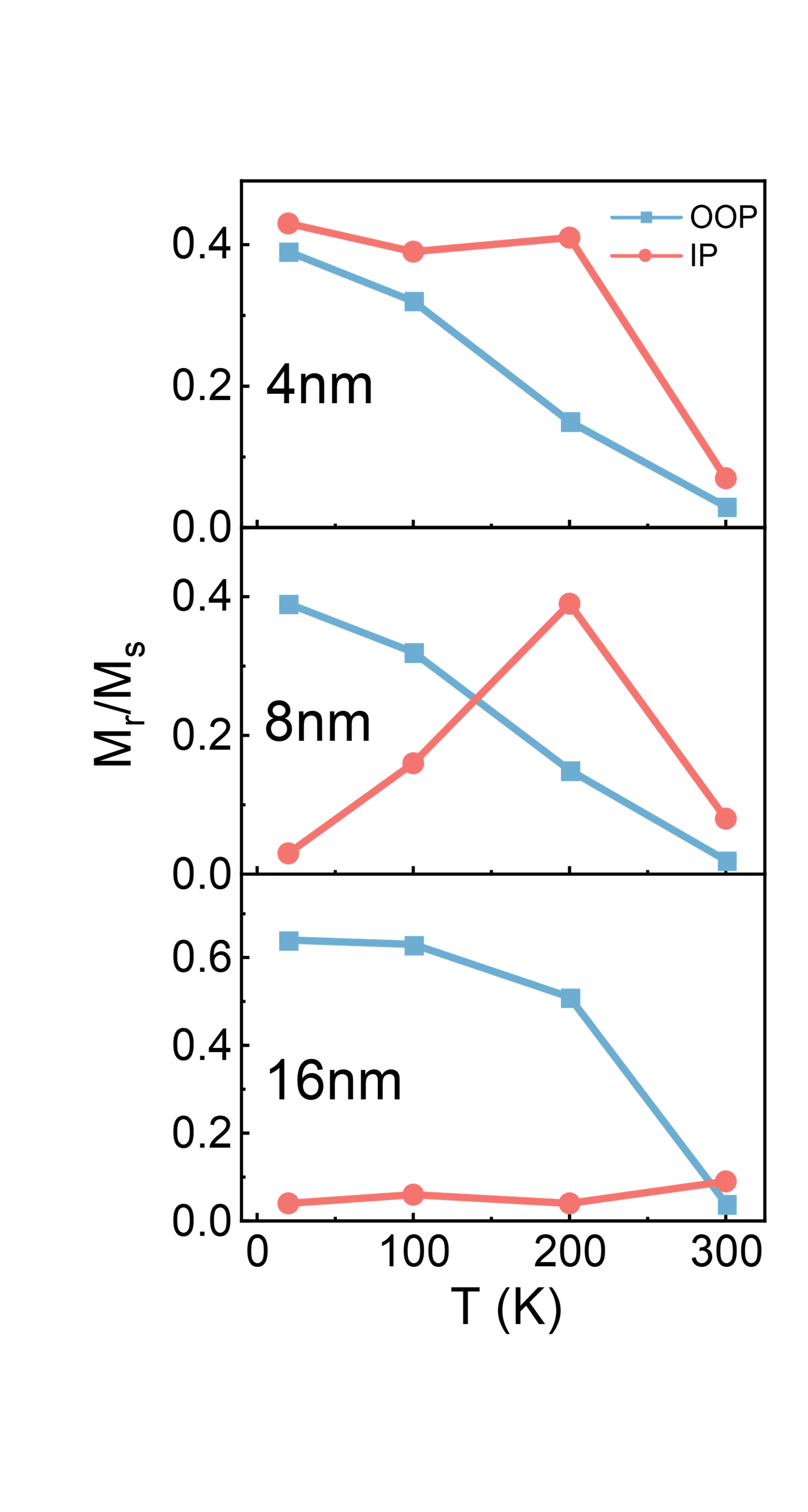


**Figure S2**. Temperature dependence of the M_r_/M_s_ ratio for 4 nm, 8 nm, and 16 nm samples under different magnetic field directions.

Section 3. Determination of Spin Reorientation Temperature from magnetization versus temperature (M-T) Curves

To directly and accurately determine the spin reorientation temperature (T_SR_) and fully understand the anisotropy evolution across all thicknesses, we performed supplementary M-T measurements. This measurement was performed using the Zero-Field-Cooled (ZFC) protocol with an applied magnetic field of 20 Oe. The magnetic field was applied along both the in-plane (H//ab) and out-of-plane (H//c) directions for the 4 nm, 8 nm, and 16 nm samples.

As shown in Figure S3, the T_SR_ can be precisely identified as the temperature at which the in-plane and out-of-plane M-T curves intersect. This direct measurement is more reliable than the indirect estimation from the effective magnetic anisotropy constant (K_eff_) versus temperature plot in the original manuscript. The results provide a clear visualization of the thickness-dependent spin reorientation behavior. For the 16 nm film, the curves cross at T_SR_ ≈ 288 K. Below this temperature, the out-of-plane magnetization is higher, indicating PMA. For the 8 nm film, the T_SR_ is significantly suppressed to ≈ 175 K. For the 4 nm film, the in-plane magnetization is dominant across the entire temperature range, and no intersection (spin reorientation) is observed.


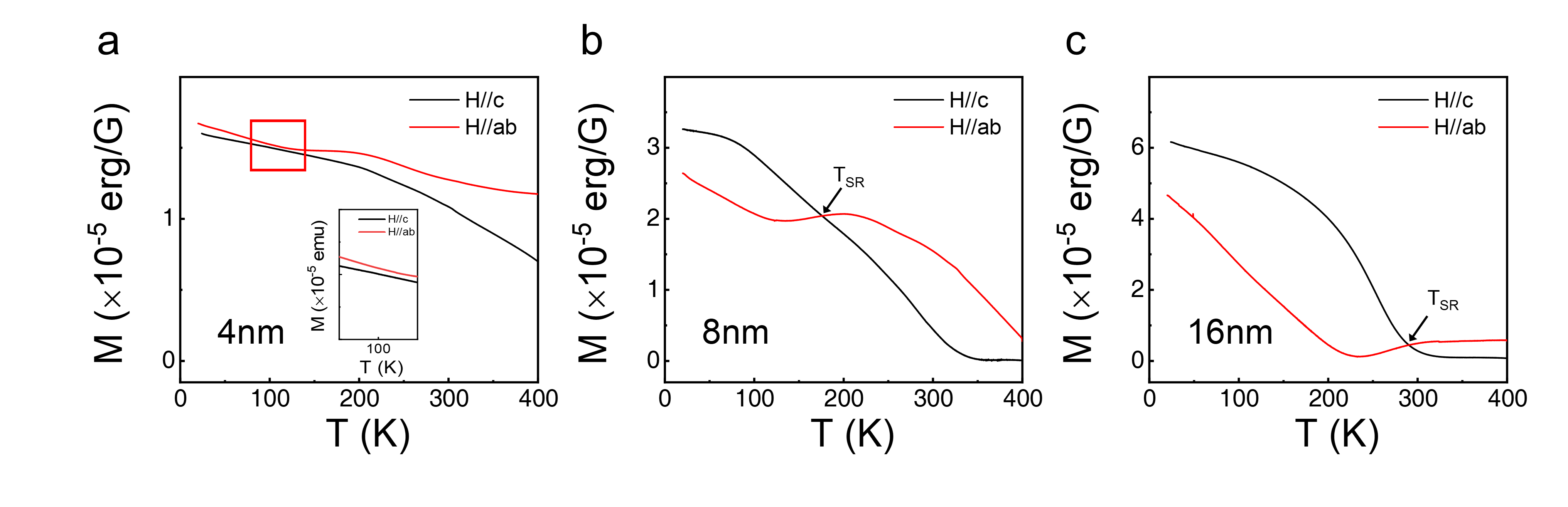


**Figure S3**. M-T curves for Fe_4_GeTe_2_ of (a) 4 nm, (b) 8 nm, and (c) 16 nm. The black and red lines represent H//c and H//ab, respectively. The measurement was performed using the ZFC protocol with an applied magnetic field of 20 Oe. The magnified region in panel (a) highlights that the in-plane and out-of-plane M-T curves for the 4 nm sample do not intersect, indicating the absence of a spin reorientation transition.

Section 4. Probing Magnetic Anisotropy via Electrical Transport in Fe_4_GeTe_2_

To further validate the magnetic testing results of the Fe_4_GeTe_2_, we performed electrical transport characterization on the Fe_4_GeTe_2_ films. We utilized the anomalous Hall effect (AHE) to probe the magnetic anisotropy electrically, a phenomenon where the anomalous Hall resistance (R_xy_) is proportional to the perpendicular component of magnetization (M_Z_). This relationship allows the electrical ratio of remanent Hall resistance to saturation Hall resistance ($\text{R}_{\text{xy}}^{\text{0}}$/$\text{R}_{\text{xy}}^{\text{s}}$) to serve as a proxy for the magnetic remanence ratio (M_r_/M_s_). Utilizing a standard Hall measurement procedure as depicted in **Figure S4**a, we measured the Hall resistance (R_xy_) of films with varying thicknesses (4 nm, 8 nm, and 16 nm) at a temperature range of 50 K to 300 K. The R_xy_-H curves are presented in Figure S4b, c, and d, respectively. All films exhibited distinct ferromagnetic characteristics within the measured temperature range, with the coercivity gradually increasing as the temperature decreased, indicating enhanced magnetic ordering. By extracting the remanent Hall resistance at zero field ($\text{R}_{\text{xy}}^{\text{0}}$) and the saturation Hall resistance ($\text{R}_{\text{xy}}^{\text{s}}$) values from the hysteresis loops, we calculated the $\text{R}_{\text{xy}}^{\text{0}}$/$\text{R}_{\text{xy}}^{\text{s}}$ ratio and examined its temperature dependence, as shown in Figure S4e. The results demonstrated that the $\text{R}_{\text{xy}}^{\text{0}}$/$\text{R}_{\text{xy}}^{\text{s}}$ ratio increased with decreasing temperature for all film thicknesses. Furthermore, at any given temperature, the thicker films (16 nm) exhibited a higher $\text{R}_{\text{xy}}^{\text{0}}$/$\text{R}_{\text{xy}}^{\text{s}}$ ratio compared to the thinner films (4 nm). This trend closely aligns with the magnetic anisotropy evolution observed in the magnetic testing.

**
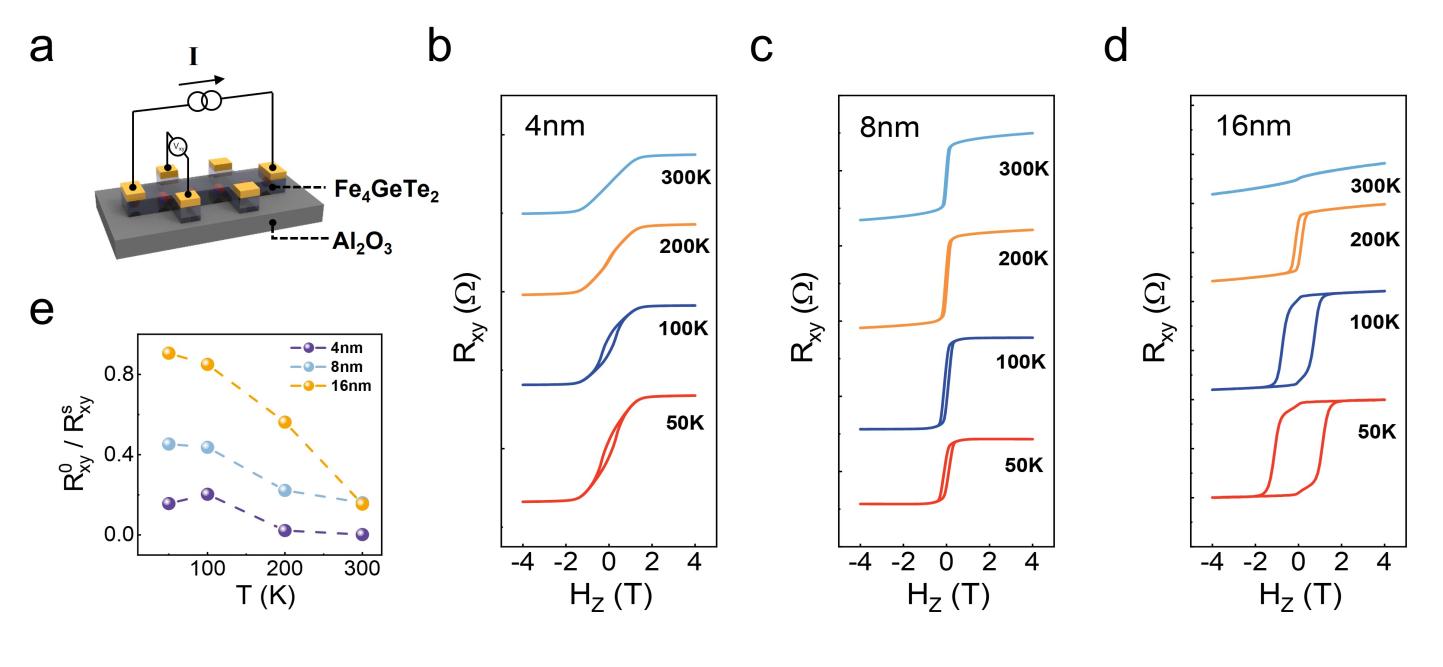
**

**Figure S4. Electrical measurement and magnetic anisotropy analysis of Fe_4_GeTe_2_ Films. a) Schematic of the Hall measurement. b) The** Hall resistance (R_xy_) versus magnetic field (H) **curves of 4 nm, c) 8 nm, and d) 16 nm Fe_4_GeTe_2_ films over a temperature range of 50 K to 300 K. e) Temperature dependence of the** remanent-to-saturation Hall resistance ratio **(**$\text{R}_{\text{xy}}^{\text{0}}$**/**$\text{R}_{\text{xy}}^{\text{s}}$**) for Fe_4_GeTe_2_ films of varying thicknesses.**

Section 5. Fe_4_GeTe_2_ RHEED and a-lattice constant analysis

**To investigate the crystal structure characteristics of Fe_4_GeTe_2_ films, we performed in-situ reflection high-energy electron diffraction (RHEED) analysis, with a particular focus on the variation of the a-lattice constant with different film thicknesses. As shown in Figure S5a, the clear streaks and sharp diffraction patterns indicate that the films possess high-quality crystallinity and a smooth surface. Using the formula a_Fe4GeTe2_ / a_Al2O3_ = D_Al2O3_ / D_Fe4GeTe2_, we roughly estimated the a-lattice constant a of Fe_4_GeTe_2_, and the results are shown in Figure S5b. As the film thickness increased to 16 nm, the Fe_4_GeTe_2_ film exhibited an a-lattice constant of approximately 4.02 Å, which is consistent with reported bulk values. Conversely, the initially grown 4 nm film showed a relatively higher a-lattice constant of approximately 4.09 Å. This represents an overall change of about 2% when compared to the 16 nm film, indicating that Fe_4_GeTe_2_ experienced slight lattice mismatch and strain on the Al_2_O_3_ substrate. The decrease in lattice constant suggests strain relaxation during growth, while the high lattice constant of the initial film may be attributed to tensile strain caused by lattice mismatch with the substrate. As the thickness increases, the structure tends to relax. The RHEED results also demonstrate that the Fe_4_GeTe_2_ films maintained good crystallinity within the studied thickness range, providing support for subsequent studies on their magnetic properties.**

**
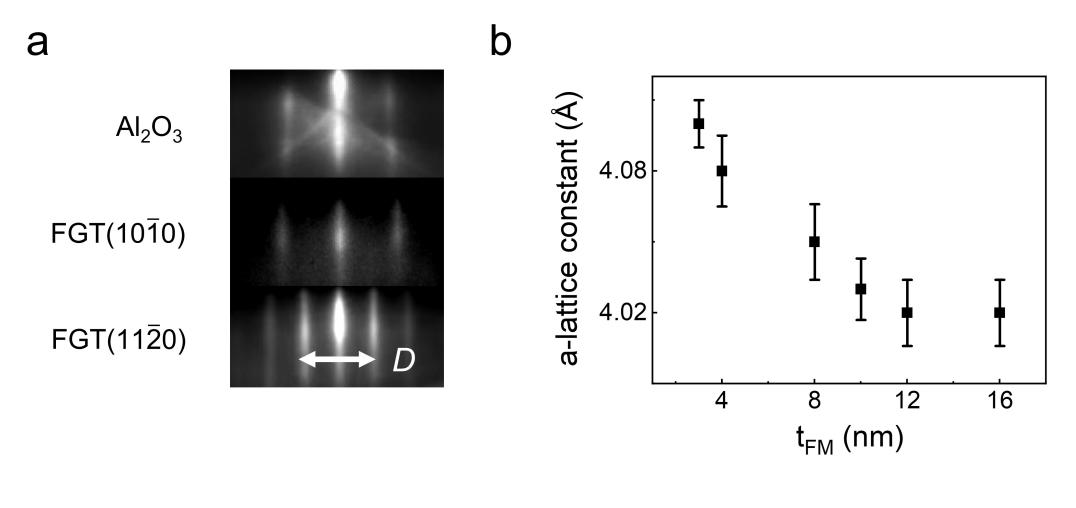
**

**Figure S5. Fe_4_GeTe_2_ RHEED pattern and a-lattice constant analysis. a) RHEED patterns of Al_2_O_3_ substrate and** **Fe_4_GeTe_2_ films. b) a-lattice constant of Fe_4_GeTe_2_ as a function of film thickness.**

Section 6. XRD Consistency Analysis of Fe_4_GeTe_2_ with the Same Thickness

**In the main text, we presented the XRD patterns of Fe_4_GeTe_2_ films with varying thicknesses and noted a low-angle shift of the (009) diffraction peak with decreasing film thickness, indicating a clear thickness dependence of the c-lattice constant. To further confirm the reliability of this conclusion, we performed XRD analysis on different samples of the same thickness to verify the consistency of the crystal structure between samples. Figure S6a, b, and c show the XRD patterns of Fe_4_GeTe_2_ films with thicknesses of 4 nm, 8 nm, and 16 nm, respectively. In each figure, we compared the XRD results of three different samples of the same thickness. As shown, the XRD patterns of samples with the same thickness are highly consistent across the entire scan range, with almost perfectly overlapping peak positions. Specifically, for the (009) diffraction peak, which is the focus of the main text, we clearly observed in the magnified insets that there is no noticeable shift in the (009) diffraction peak positions between different samples of the same thickness.**

**These results demonstrate that Fe_4_GeTe_2_ films of the same thickness obtained under identical preparation conditions possess highly consistent crystal structures. The significant shift in the (009) diffraction peak position is observed only between samples of different thicknesses, further confirming that the thickness dependence of the c-lattice constant is an intrinsic characteristic induced by the Fe_4_GeTe_2_ /Al_2_O_3_ interface.**

**
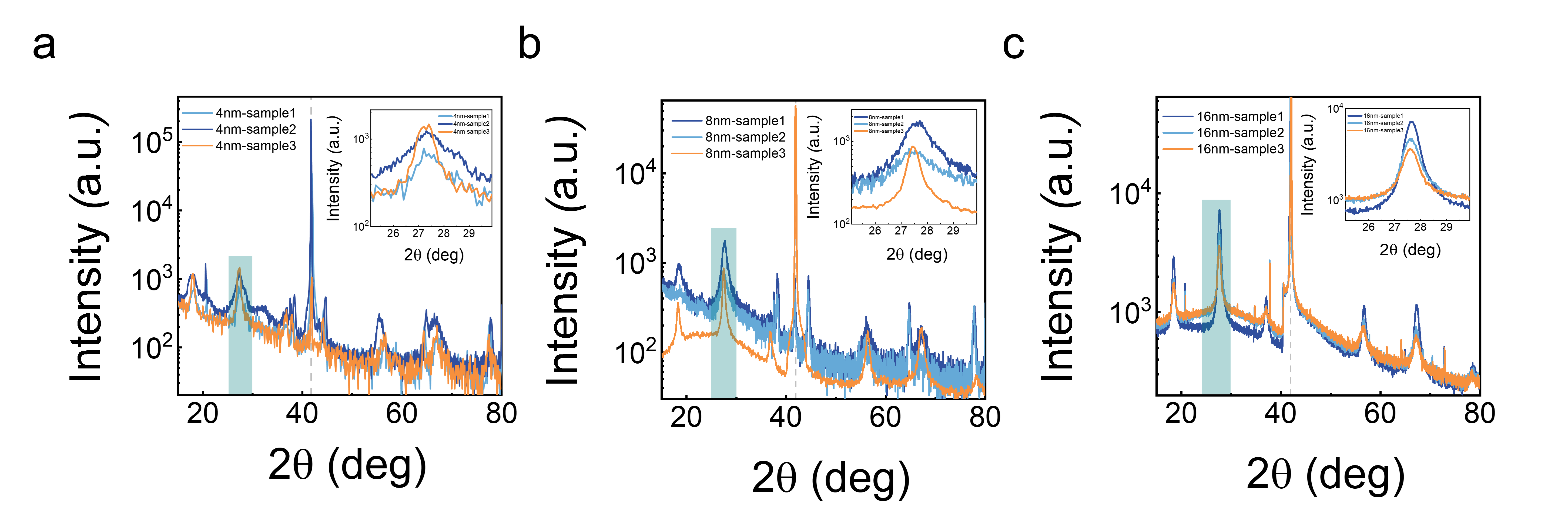
**

**Figure S6. XRD Peak Consistency Analysis of Fe_4_GeTe_2_ Films of the Same Thickness. a) XRD patterns of 4 nm, b) 8 nm, and c) 16 nm thick Fe_4_GeTe_2_ films. Insets show magnified views of the (009) diffraction peak.**

Section 7. Van der Waals gap measurements of 8nm Fe_4_GeTe_2_

To further investigate the thickness dependence of the interlayer van der Waals gap (d_Gap_) in Fe_4_GeTe_2_, we extended our analysis to an 8 nm thick sample. **Figure S7** presents the HAADF-STEM images of the 8 nm Fe_4_GeTe_2_, specifically focusing on two distinct regions: four layers near the interface (1L-4L) and four layers away from the interface (5L-8L). To ensure accurate measurements and minimize errors, we aligned the bottommost atomic layers of the selected image segments. Within these regions, five distinct analysis areas (labeled I-V) were selected and marked with colored rectangular boxes. These areas were chosen to capture the d_Gap_ variations.

The d_Gap_ were precisely measured within each of the five selected analysis areas for each layer. The results of these measurements are summarized in **Table S1**. Consistent with the observations in the 10 nm Fe_4_GeTe_2_ (discussed in the main text), the 8 nm sample also exhibits a trend of decreasing d_Gap_ with increasing distance from the interface. Specifically, the layers near the interface (1L-4L) show a significant expansion in d_Gap_ compared to the layers further away (5L-8L).

**
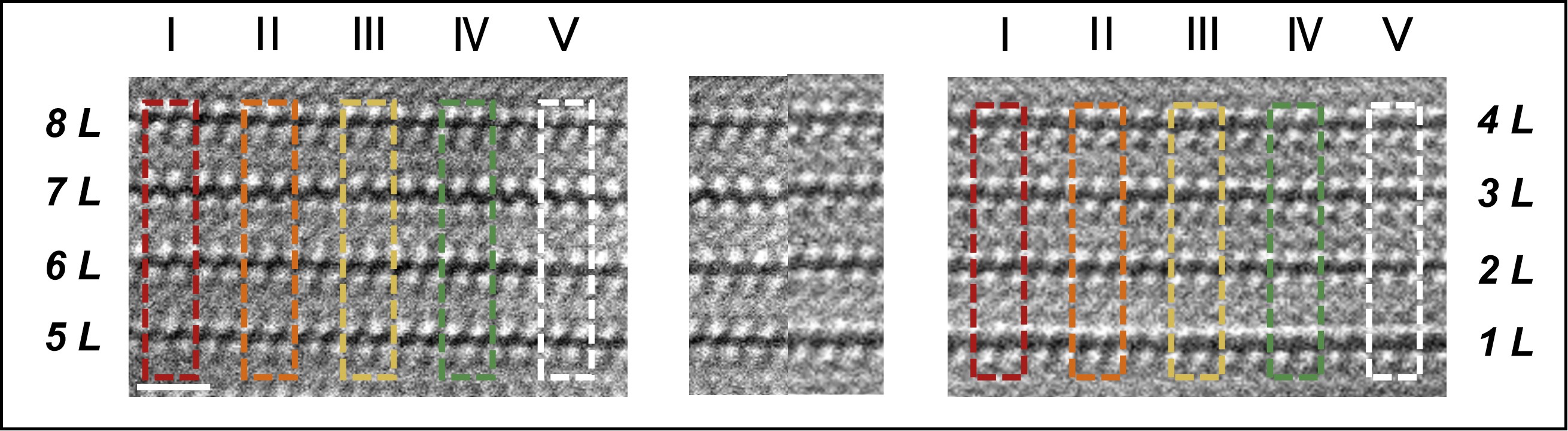
**

**Figure S7.** HAADF-STEM image of four layers near the interface (1L-4L) and four layers away from the interface (5L-8L) in 8 nm Fe_4_GeTe_2_. The middle image shows a detailed d_Gap_ comparison. Colored rectangles mark five selected analysis regions (I-V) for measuring the d_Gap_. Scale bar: 1 nm.

**Table S1. Interlayer van der Waals Gap Measurements (Å)**

|  | $\text{D}_{\text{Ⅰ}}$ | $\text{D}_{\text{Ⅱ}}$ | $\text{D}_{\text{Ⅲ}}$ | $\text{D}_{\text{Ⅳ}}$ | $\text{D}_{\text{Ⅴ}}$ | $\bar{\text{D}}$ |
| --- | --- | --- | --- | --- | --- | --- |
| 8L | 2.75 | 2.75 | 2.70 | 2.65 | 2.51 | 2.67 |
| 7L | 2.75 | 2.80 | 2.80 | 2.80 | 2.75 | 2.78 |
| 6L | 2.70 | 2.80 | 2.80 | 2.80 | 2.75 | 2.78 |
| 5L | 2.70 | 2.70 | 2.70 | 2.80 | 2.75 | 2.73 |
| 4L | 2.80 | 2.80 | 2.80 | 2.75 | 2.75 | 2.78 |
| 3L | 2.85 | 2.89 | 2.85 | 2.85 | 2.85 | 2.86 |
| 2L | 3.04 | 3.04 | 3.04 | 2.99 | 2.94 | 3.01 |
| 1L | 3.28 | 3.28 | 3.28 | 3.04 | 3.28 | 3.23 |

**Section 8. Raman Spectroscopy Analysis of Fe_4_GeTe_2_** **with different thicknesses**

We performed Raman spectroscopy on the 4 nm and 16 nm Fe_4_GeTe_2_ films. As a technique highly sensitive to interlayer coupling and lattice strain, Raman spectroscopy can provide strong corroborating evidence for our structural analysis. We chose these two thicknesses because they represent the extremes in our study and can most clearly highlight the structural evolution trend caused by interface effects. The Raman spectroscopy, as shown in **Figure S8**, display two characteristic phonon modes of layered materials: the out-of-plane A_z_ mode and the in-plane E_y_ mode, which are located at 122 cm^-1^ and 138 cm^-1^ respectively for the 16 nm film, are highly consistent with previous reports^[1]^. It is important to note that specific theoretical studies on the phonon modes of Fe_4_GeTe_2_ are currently unavailable. However, we performed the mode assignment based on the structural similarities within the Fe_n_GeTe_2_ family. The FGT compounds share a highly similar local chemical environment for the Te atoms at the vdW interface. This is supported by existing experimental data showing that the characteristic out-of-plane Te vibrational peak positions in Fe_3_GeTe_2_ and Fe_5_GeTe_2_ are essentially identical^[2-3]^. Therefore, we believe that making a tentative assignment based on the conserved local vdW interface structure is the most reasonable approach currently available. As the film thickness decreases from 16 nm to 4 nm, a significant redshift of the A_z_ mode is observed, from 122 cm^-1^ down to 104 cm^-1^. This is a direct signature of weakened interlayer vdW coupling^[4]^ and is in full agreement with the vdW gap expansion we observed via HRTEM and XRD.


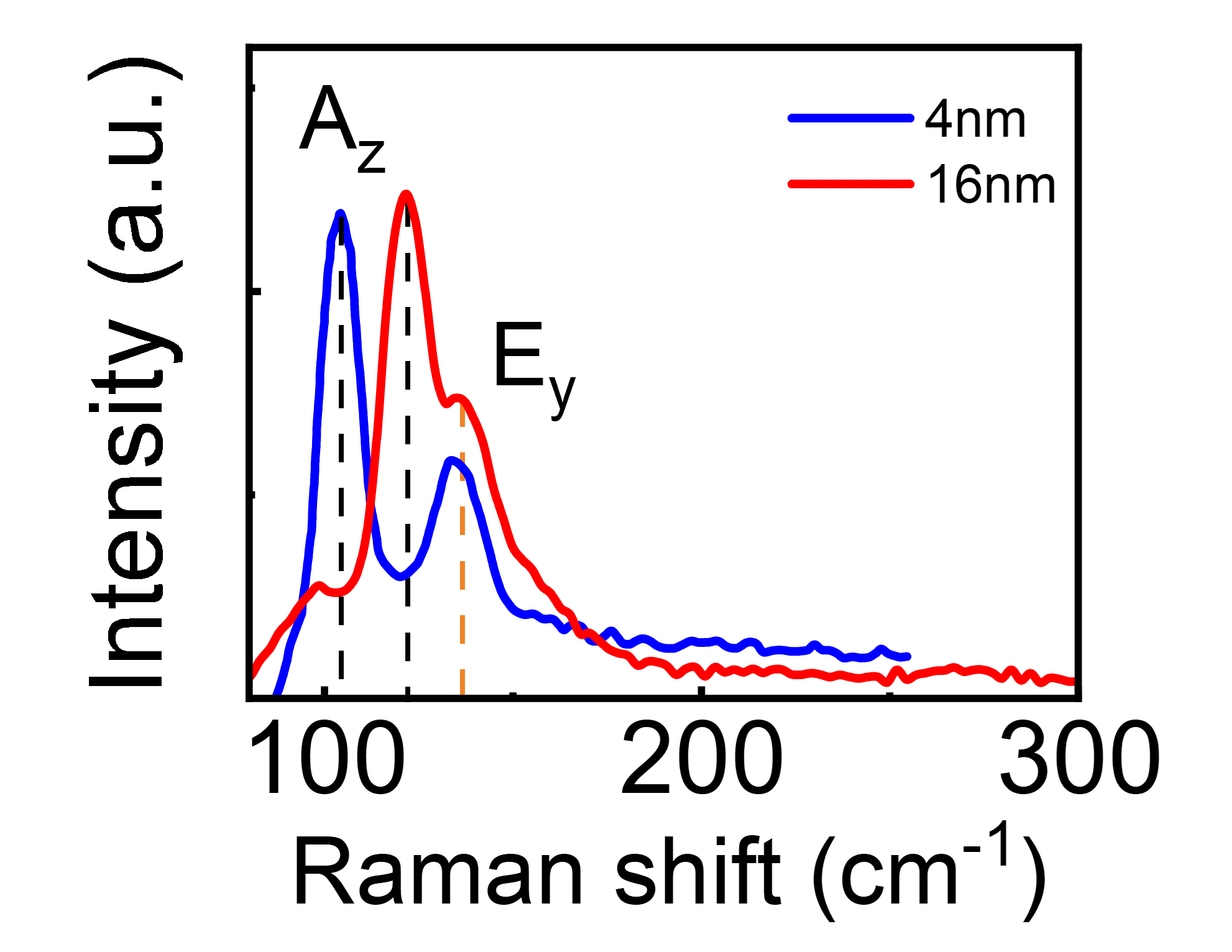


**Figure S8**. Raman spectra of the 4 nm and 16 nm Fe_4_GeTe_2_. The two prominent peaks is the out-of-plane A_z_ mode and the in-plane E_y_ mode.

Section 9. First-principles calculations for bulk Fe_4_GeTe_2_

As shown in **Figure S9**, the atomic structure of bulk Fe_4_GeTe_2_ exhibits an ABC stacking pattern with a three-layer periodicity along the c-axis, separated by vdW gaps. Within each monolayer, Fe atoms occupy two inequivalent Wyckoff sites, α-Fe and β-Fe, exhibiting distinct magnetic moments. Our calculations show that the spin at α-Fe is 3/2, and at β-Fe is 1, yielding an average magnetic moment of 2.5 μB per Fe atom at 0 K, consistent with previously reported values. To determine the magnetic easy axis, we evaluated the MAE of Fe_4_GeTe_2_ by orienting the spin axis along various directions. The MCA, calculated by considering spin-orbit coupling (SOC), is approximately 2.18 meV/f.u. The MSA, arising from magnetic dipole-dipole interactions, is -0.082 meV/f.u. These results confirm that bulk Fe_4_GeTe_2_ favors an out-of-plane spin orientation, consistent with experimental results.


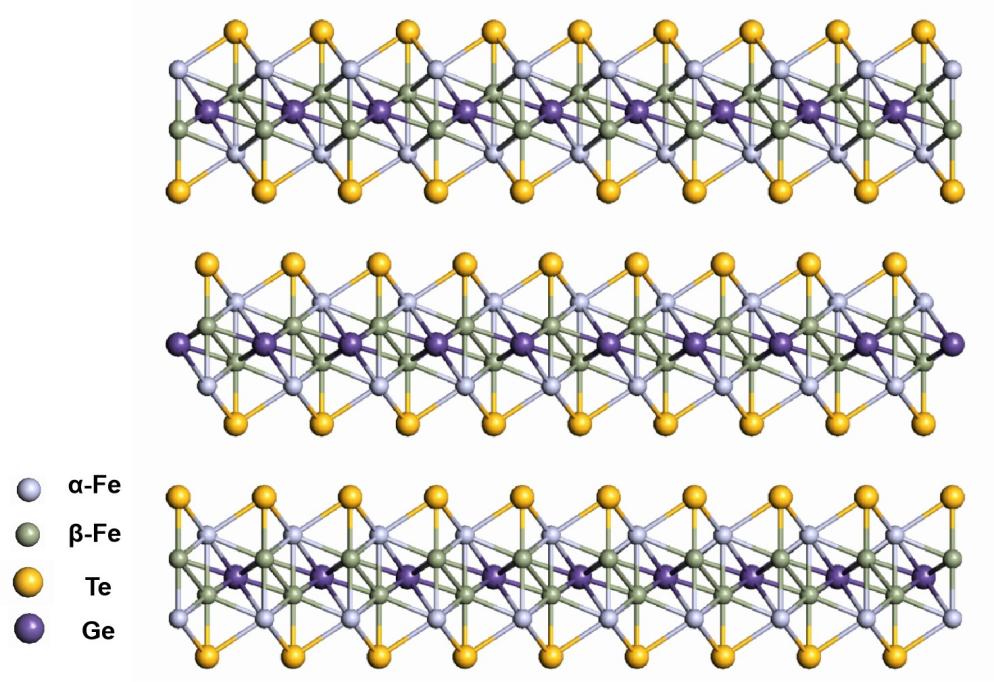


**Figure S9. Atomic structure of bulk Fe_4_GeTe_2_.** Showing two types of Fe atoms with different symmetric positions and spins, labeled as α-Fe (grey) and β-Fe (green).

Section 10. First-principles calculations of in-plane strain effects on magnetic anisotropy of Fe_4_GeTe_2_

**To investigate the influence of in-plane strain on the magnetic anisotropy of Fe_4_GeTe_2_, we performed first-principles calculations by applying biaxial strain along the a-axis to the bulk Fe_4_GeTe_2_ structure. The strain ε is defined as the relative change of the lattice constant with respect to the equilibrium value. We varied ε from -4% (compressive strain) to +4% (tensile strain) and calculated the corresponding magnetocrystalline anisotropy energy (MCA), magnetic shape anisotropy energy (MSA), and total magnetic anisotropy energy (MAE). The results are presented in Figure S10. It is evident that within the investigated strain range, Fe_4_GeTe_2_ retains its preference for perpendicular magnetic anisotropy (PMA). Both the MCA and total MAE remain positive, indicating that the out-of-plane magnetization direction is energetically favorable. Moreover, the MSA remains relatively stable and close to zero across the applied strain range.**


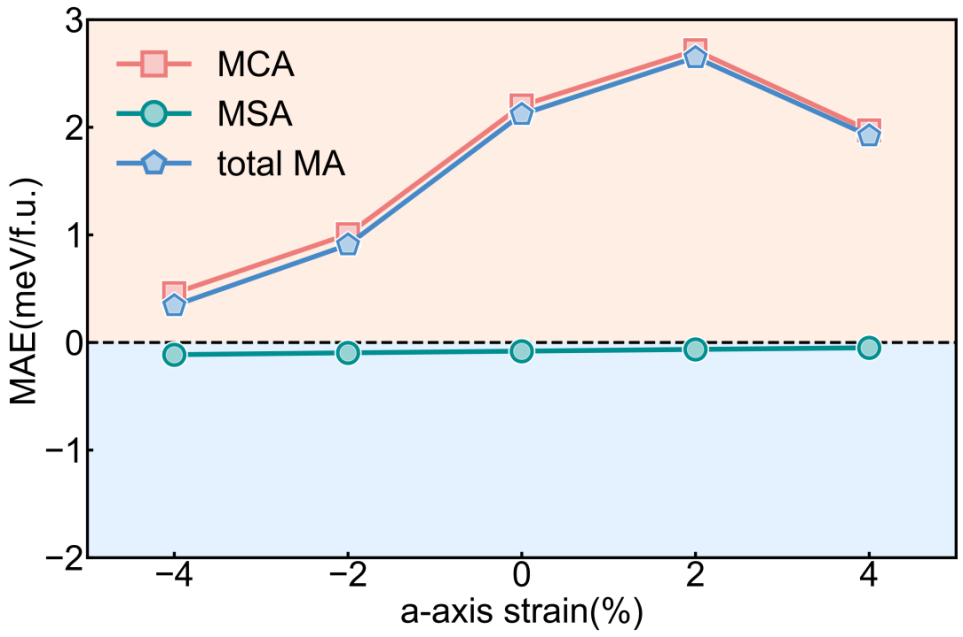


**Figure S10. Magnetic anisotropy as a function of a-axis biaxial strain in Fe_4_GeTe_2_.**

Section 11. Analysis of the Thickness Dependence of Shape Anisotropy

**The shape anisotropy energy density (K_MSA_), which arises from the demagnetizing effect, provides a driving force for in-plane magnetization. To precisely evaluate the direct impact of thickness on this term, we performed additional first-principles (DFT) calculations of the MSA energy for Fe_4_GeTe_2_ slabs of 2, 3, 4, and 5 layers, while keeping the vdW gap constant. As shown in **Figure S11**, The results confirm that decreasing the film thickness leads to only a slight enhancement in MSA (from –0.240 meV/f.u. for the 5-layer slab to –0.247 meV/f.u. for the 2-layer slab). This negligible change is two orders of magnitude smaller than the dramatic change in MCA (on the order of several meV) caused by the vdW gap expansion, as discussed in the main text. This analysis confirms that the dominant physical mechanism for the observed enhancement of in-plane anisotropy is the pronounced suppression of MCA, rather than the thickness dependence of MSA.**


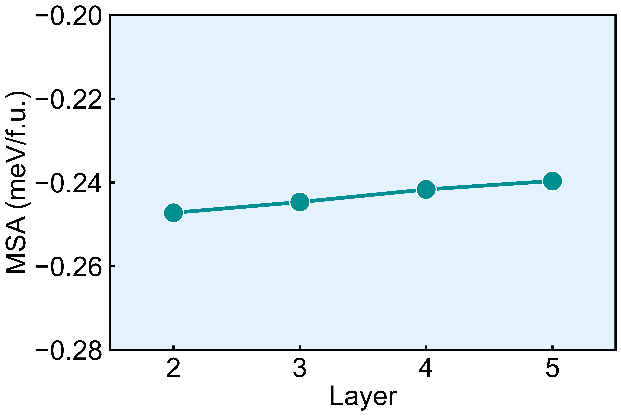


**Figure S11. Layer dependence of the MSA energy for Fe_4_GeTe_2_ slabs.**

Section 12. Units and Estimation of MSA

The MAE is determined from first-principles calculations as the sum of the MCA and MSA contributions:

MAE = MCA + MSA

In this work, the calculated MAE is expressed in units of millielectron volts per formula unit (meV/f.u.). This unit is employed to isolate the intrinsic effects of vdW gap engineering on the Fe_4_GeTe_2_ unit cell. By normalizing the energy to the formula unit, any extrinsic contributions arising from changes in the total cell volume during vdW gap expansion are excluded. This method facilitates a direct, volume-independent comparison of anisotropy contributions under varying structural conditions.

For comparison with experimental literature, these values can be converted to the conventional unit of Joules per cubic centimeter (J/cm^3^) using the following relation:

$$\text{E[J/}\text{cm}^{\text{3}}\text{]=E[meV/f.u.]×}\frac{\text{1.602×}\text{10}^{\text{-22}}\text{J/meV}}{\text{V}_{\text{f.u.}}\text{[}\text{cm}^{\text{3}}\text{]}}$$

where $V_{f.u.}$ is the volume of the formula unit obtained from the optimized bulk structure. Furthermore, the MSA was estimated from experimental data for comparison with theoretical calculations. It should be noted that the first-principles calculations are performed at 0 K, whereas the experimental measurements are conducted at a finite low temperature. Due to the strong temperature dependence of M_S_, a direct quantitative comparison between the two may be subject to deviation. For an infinite thin film, the MSA density can be estimated by the formula E_MSA_=2πM_S_^2^. For instance, using the experimentally measured saturation magnetization (M_S_ ~ 800 erg/(G·cm^3^)) for the 4 nm sample at 20 K, the calculated E_MSA_ is approximately -0.11 meV/f.u. after unit conversion. This estimated result is of the same order of magnitude as the theoretically calculated values (approximately -0.08 to -0.12 meV/f.u.), which verifies the consistency between them.

**Reference**

[1] H. Yu, X. Li, Y.-Q. Bie, L. Yan, L. Zhou, P. Yu, G. Yang, *Nature Communications* **2025**, 16, 7698.

[2] J. Liang, S. Liang, T. Xie, A. F. May, T. Ersevim, Q. Wang, H. Ahn, C. Lee, X. Zhang, J.-P. Wang, M. A. McGuire, M. Ouyang, C. Gong, *Physical Review Materials* **2023**, 7, 014008.

[3] L. Du, J. Tang, Y. Zhao, X. Li, R. Yang, X. Hu, X. Bai, X. Wang, K. Watanabe, T. Taniguchi, D. Shi, G. Yu, X. Bai, T. Hasan, G. Zhang, Z. Sun, *Advanced Functional Materials* **2019**, 29, 1904734.

[4] S. Huang, L. Liang, X. Ling, A. A. Puretzky, D. B. Geohegan, B. G. Sumpter, J. Kong, V. Meunier, M. S. Dresselhaus, *Nano Letters* **2016**, 16, 1435.
